# The Role of Secondary Brain Insults in Status Epilepticus: A Systematic Review

**DOI:** 10.3390/jcm9082521

**Published:** 2020-08-05

**Authors:** Candice Fontaine, Gwenaelle Jacq, François Perier, Mathilde Holleville, Stephane Legriel

**Affiliations:** 1 Medical-Surgical Intensive Care Unit, Hopital Paris Saint Joseph, 185 Rue Raymond Losserand, 75014 Paris, France; candicefontaine@yahoo.com; 2 IctalGroup, 177 rue de Versailles, 78150 Le Chesnay CEDEX, France; gjacq@ch-versailles.fr (G.J.); francoisperier@hotmail.fr (F.P.); mathildeholleville@gmail.com (M.H.); 3 Intensive Care Department, Centre Hospitalier de Versailles, 177 rue de Versailles, 78150 Le Chesnay CEDEX, France; 4 Medical Intensive Care Unit, Assistance Publique-Hôpitaux de Paris, CHU Henri Mondor, 51 Avenue du Maréchal de Lattre de Tassigny, 94010 Créteil, France; 5 Department of Anaesthesiology and Critical Care, Hôpitaux Universitaires Paris Nord Val de Seine, Hôpital Beaujon, 100 Boulevard du Général Leclerc, 92110 Clichy, France; 6 UVSQ, INSERM, University Paris-Saclay, CESP, Team «PsyDev», 94800 Villejuif, France

**Keywords:** status epilepticus/complications, status epilepticus/etiology, status epilepticus/therapy, outcome, intensive care unit, humans, animal models

## Abstract

(1) Background: Little is known about the impact of pathophysiological mechanisms that underlie the enhancement of excitotoxicity and the neuronal consequences of status epilepticus (SE), as well as the clinical consequences of secondary brain insults (SBI) in patients with SE on outcome; (2) Methods: Electronic searches were conducted in May 2020 using Medline via PubMed, Embase, and Google Scholar (#CRD42019139092). Experimental studies of animals or randomized, observational, controlled trials of patients with SE in indexed journals were included. There were no language or date restrictions for the published literature included in this review. Information was extracted on study design, sample size, SBI characteristics, and primary and secondary outcomes, including the timing of evaluation; (3) Results: Among the 2209 articles responding to our inclusion criteria, 56 were included in this systematic review. There are numerous experimental data reporting the deleterious effects associated with each of the SBI in animals exposed to SE. In humans, only the effect of target temperature management in hypothermia (32–34 °C) has been explored. (4) Conclusions: There is little experimental evidence that favors the control of secondary brain insult after SE. Further studies are required to assess the neuroprotective interest of secondary brain insult control after SE in humans.

## 1. Introduction

Status epilepticus (SE) is a major medical condition that is associated with poor outcome in approximately 50% of cases, despite the use of conventional anticonvulsive treatments. The age of the patients, a previous history of epilepsy, SE refractoriness, and a primary cerebral insult as the cause of SE have been identified as independent predictors of poor outcome [1,2,3].

Secondary brain insults refer to a cascade of cellular, biochemical, tissue, or blood vessel changes that occur over a period ranging from minutes to days after a primary brain injury caused by various conditions, such as traumatic brain injury, stroke, or subarachnoid hemorrhage, ultimately resulting in additional brain tissue damage [4,5]. The common denominator for all factors of secondary brain insults is cerebral blood flow (CBF) which can be approximated by monitoring cerebral perfusion pressure (CPP) [6]. Several factors can affect CBF: flow-metabolism coupling and cerebral autoregulation/mean arterial blood pressure, arterial partial pressure of carbon dioxide (PaCO_2_), arterial partial pressure of oxygen (PaO_2_), temperature, natremia and osmolality, glycemia, and hemoglobinemia. The occurrence of these irreversible secondary brain lesions has an impact on outcome, not only in terms of mortality, but also on longer-term functional neurological impairment [7,8,9,10,11,12]. 

In SE, an experimental study performed on baboons exposed to prolonged epileptic seizures showed physiological changes to be characterized by a two-step phenomenon. First, animals demonstrated adaptive adrenergic activation as a mechanism to protect the brain by increasing CBF and thus improving oxygen and glucose delivery during the acute phase of seizure activity. Second, the animals demonstrated an inadequate decrease in CBF and a deleterious reduction of oxygen and glucose delivery to the brain as the seizures continued, associated with several systemic factors that correspond to secondary brain insults, also reported in primary brain injury, from other causes: mild arterial hypotension, acidosis, hypoxia, hyperpyrexia, and severe hypoglycemia [13,14,15]. 

The aim of this systematic review was to highlight important aspects of secondary brain insults in the population of patients with SE.

## 2. Experimental Section

Data reporting in this systematic review of secondary brain insults and SE is in accordance with the recommendations included in the PRISMA statement and the study protocol was previously recorded in PROSPERO (#CRD42019139092).

### 2.1. Definitions

In humans, SE has been defined, according to the “Report of the ILAE Task Force on the Classification of Status Epilepticus”, as an ongoing clinical and/or electroencephalographic seizure activity “resulting either from the failure of the mechanisms responsible for seizure termination or from the initiation of mechanisms, which lead to abnormally prolonged seizures (after time point T_1_). It is a condition, which can have long-term consequences (after time point T_2_), including neuronal death, neuronal injury, and alteration of neuronal networks, depending on the type and duration of seizures”. Accordingly, tonic-clonic SE, focal SE with impaired consciousness, and absence SE were considered in cases of seizure lasting more than 5 min, 10 min, and 10–15 min, respectively [16].

Secondary brain insults are defined as factors that may affect CBF in various situations of primary brain injury: flow-metabolism coupling and cerebral autoregulation/mean arterial blood pressure (MAP), arterial partial pressure of carbon dioxide (PaCO_2_), arterial partial pressure of oxygen (PaO_2_), temperature, natremia and osmolality, glycemia, or hemoglobinemia [7]. 

### 2.2. Eligibility Criteria

This review focused on studies describing and evaluating three types of outcome predictors: neuronal damage, excitotoxicity, and clinical outcomes. All studies including patients or animals who experienced SE were considered for inclusion. Patients or animals with imprecise seizure duration or not fulfilling the criteria for SE were excluded from the review process. Patients or animals with postanoxic SE were also excluded. Experimental studies in which the outcome was described as neuronal damage or excitotoxicity (seizure frequency, duration, and latency to onset) were retained for inclusion. Prognostic studies in which the neurological outcome was described using either the Glasgow Outcomes Scale (GOS) or modified Rankin Scale (mRS), were included in the review. A favorable outcome was defined as a GOS score of 4 or 5, or an mRS score of 1 to 3. Outcome studies were included if the patients were assessed at hospital discharge, or ≥3 months after discharge, when available.

### 2.3. Search Strategy

We searched MEDLINE via PubMed, EMBASE, and GOOGLE SCHOLAR using the following terms: “status epilepticus (MeSH Terms)” AND terms corresponding to the various secondary brain insults (Supplemental file). Two investigators (Stephane Legriel, MD, PhD and Candice Fontaine, MD) independently assessed the eligibility of all studies identified in the initial search. Disagreements were resolved by consensus between the two investigators.

**Figure 1:**
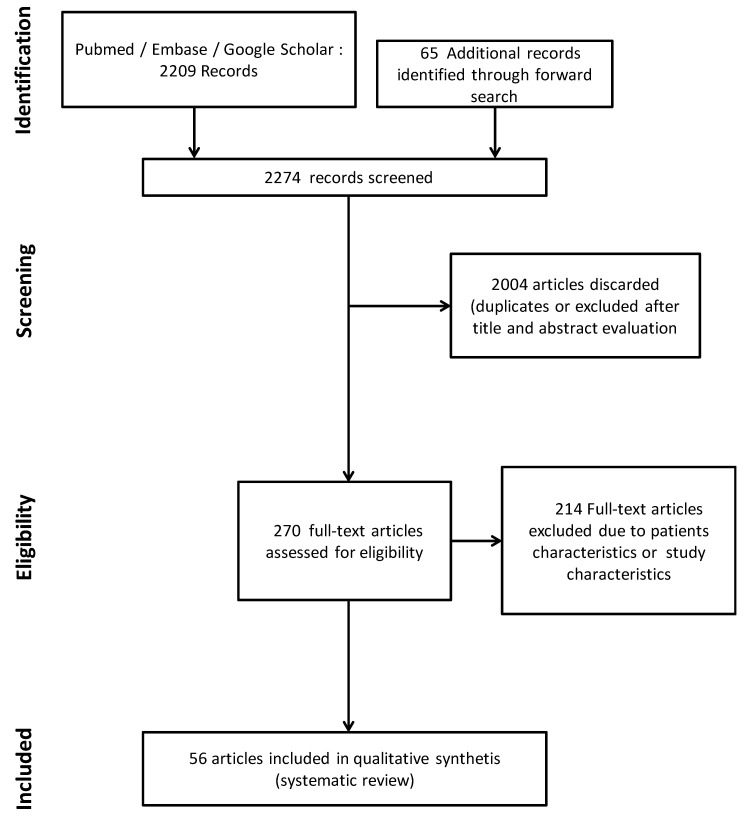
Flow chart of study selection.

All citations of identified articles were downloaded for elimination of duplicates and further abstract analysis and article selection. Full texts of all remaining citations were downloaded and carefully checked for inclusion. We activated an automatic PubMed alert system from the first article selection to the last search round, performed on May 2020 to maintain an updated search strategy. The reference lists of relevant review articles and articles selected for inclusion in this review were searched manually for other potential studies to ensure that all potentially relevant articles were included (Figure 1).

### 2.4. Study Selection and Data Extraction

Experimental studies of animals or randomized, observational, controlled trials of patients with SE in indexed journals were included. There were no language or date restrictions for the published literature included in this review. Studies were selected and screened by titles and abstracts to identify studies reporting any of the selected interventions and outcomes of focus. Data extraction was performed using a dedicated form. The following data were extracted for each study: first author, publication year, study design, sample size, secondary brain insult characteristics, and primary and secondary outcomes, including the timing of evaluation.

## 3. Results

Among the 2209 articles responding to our search strategy, 56 were included in this systematic review (Figure 1). Study results covered experimental data showing the interplay between various physiologic derangements indistinguishable from the effects of ongoing seizure activity and from the effects of physiology on seizures, and the resulting secondary brain insults. The only clinical data were covered by a randomized controlled trial focusing on the effect of temperature.

### 3.1. Experimental Data

**Figure 2:**
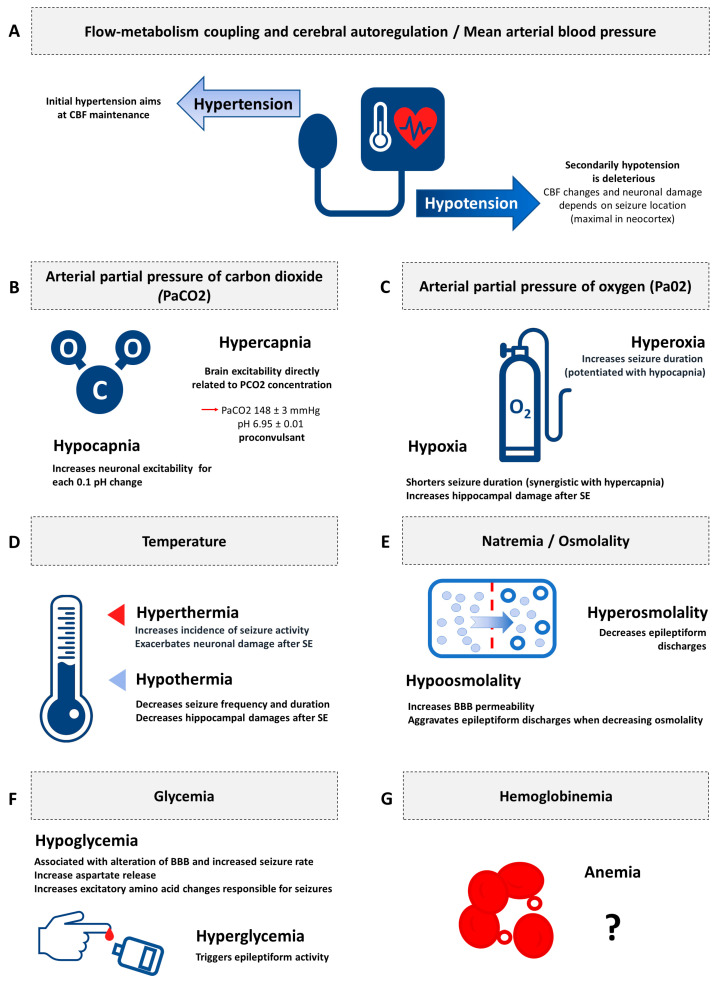
Synthesis of the evidence of secondary brain insults in experimental status epilepticus. CBF: cerebral blood flow; PaCO_2_: arterial partial pressure of carbon dioxide; PaO_2_: arterial partial pressure of oxygen; BBB: blood brain barrier. (**A**) Flow-metabolism coupling and cerebral autoregulation/Mean arterial blood pressure; (**B**) Arterial partial pressure of carbon dioxide (PaCO2); (**C**) Arterial partial pressure of oxygen (Pa02); (**D**) Temperature; (**E**) Natremia/Osmolality; (**F**) Glycemia; (**G**) Hemoglobinemia.

Figure 2 illustrates the synthesis of the evidence of secondary brain insults in experimental status epilepticus.

#### 3.1.1. Flow-Metabolism Coupling and Cerebral Autoregulation/Mean Arterial Blood Pressure

The natural course of SE is characterized by an initial phase of adrenergic activation [17,18], resulting in an increase in CBF, characterized by the association of arterial blood pressure hypertension and a decrease in cerebrovascular resistance. [19,20,21] The second phase consists of a progressive decrease in arterial blood pressure, and finally, hemodynamic failure, responsible for a deleterious drop in CBF [15,20,22]. 

#### 3.1.2. Arterial Partial Pressure of *Carbon Dioxide*

Hypocapnia is routinely used as an activation test during electroencephalogram recording [23]. Several clinical studies reported an association between respiratory alkalosis and seizure activity [24,25,26,27,28], potentiated by tapering antiepileptic drugs [28]. Paradoxically, in a large retrospective study, a clinical seizure was elicited after 5 min of hyperventilation in only 2/433 (0.5%) of hospitalized patients monitored by long-term video-EEG in an epilepsy unit [24]. However, various experimental findings support the hypothesis of enhancing neuronal excitability after the induction of hypocapnia [23]. In a rodent model of epilepsy, hypocapnia increased ictal activity (population spike amplitude, spreading depression-like response, postsynaptic neurons electrical excitability), which varied for each decrease in pH of 0.1 [29]. Seizure duration was demonstrated to be inversely related to PaCO_2_ levels in another study of dogs receiving electroconvulsive therapy [30]. 

Studies to assess the pro- or anti-convulsant properties of hypercapnia yielded conflicting results. An increase in PaCO_2_ was associated with the stimulation of inhibitory interneurons which halted seizure activity in several experimental studies [31,32]. However, seizure activity has been associated with respiratory acidosis in human [17,33], and some experimental data support the hypothesis of carbon dioxide neurotoxicity. Woodbury et al. [34] and Withrow et al. [35] reported seizure activity in all rats exposed to an atmosphere containing 45% CO_2_ and 90% of adult rats exposed to 30% CO_2_ for 8 min, respectively. The relationship between the CO_2_ concentration in inspired air, plasma PaCO_2_, and brain excitability was demonstrated by Brodie et al. Indeed, rats exposed to an atmosphere containing 30% CO_2_ (PaCO_2_ 148 ± 3 mmHg, pH 6.95 ± 0.01) exhibited intermittent, repetitive, clonic seizures, whereas rats exposed to concentrations of 50% CO_2_ (PaCO_2_ 264 ± 48 mmHg, pH 6.72 ± 0.02) did not exhibit seizures but appeared to be anesthetized [34]. The effect of CO_2_ on brain excitability appears to be directly related to the concentration inhaled, thus the PaCO_2_ and the level of acidosis. The underlying mechanism of seizures in hypercapnia has yet to be completely understood. In an experimental setting, Katsura et al. induced cerebral ischemia by elevating the CO_2_ concentration in inhaled gas, reaching PaCO_2_ values of 300 mmHg. Protein kinase systems have been suggested to be involved in neurotransmission and affected by acidosis. This is one potential mechanism that may induce seizure activity during hypercapnia, but there are no data to support this hypothesis [36]. 

#### 3.1.3. Arterial Partial Pressure of Oxygen

Hypoxia has been shown to shorten the duration of seizures produced during kainic acid-induced SE, but was associated with higher hippocampal neuronal toxicity, illustrating the potentiation of neuronal damage by hypoxia during long-lasting seizures [37]. Another study found a synergistic association between hypoxemia and hypercapnia to shorten seizure duration [38]. An interventional therapeutic approach was evaluated by Wasterlain et al., who tested the interest of mechanical ventilation and muscular blockade in 10 rats receiving repetitive convulsive electroshocks. They found that the correction of hypoxemia by supportive techniques markedly diminished mortality relative to a control group of hypoxemic rats [39]. However, the correction of hypoxia alone was not sufficient to avoid neuronal toxicity, since this damage can result from SE per se, with or without its association with tissue hypoxia [40]. Finally, preconditioning hypoxemia was the only situation found with a favorable outcome. Application of preconditioning hypoxia to rats with kainic acid-induced SE demonstrated adaptive properties, leading to better control of seizure activity [41] and neuroprotection by decreasing brain edema [42]. 

Hyperoxia-induced seizures are a well-known complication, in particular during hyperbaric oxygen administration [43,44]. However, demonstration of neuronal toxicity of hyperoxia in normobaric conditions of administration in the context of SE is still a subject of debate. In animals, as in humans, hyperoxia increases seizure duration, especially as it is associated with hypocapnia [30,38]. Given these findings, epileptic rats mechanically ventilated with 100% oxygen, surprisingly, did not demonstrate neuronal damage that was different from that of normoxic epileptic animals [45,46]. 

#### 3.1.4. Temperature

Fever is the most studied cause of secondary brain insult in SE [47]. Indeed, hyperthermia between 39.5 and 42 °C was associated with an increased incidence of seizure activity and exacerbated neuronal damage in studies of rats with self-sustaining SE [48,49,50]. Conversely, mouse models exposed to SE and subsequently treated showed a strengthening of the anesthetic’s antiepileptic activity after induction of hypothermia, characterized by a decrease in the frequency and latency to the onset of seizures and a decrease in recurrence after withdrawal of the anesthetic treatments [49,51,52,53]. Moreover, the induction of SE in rats under conditions of normothermia or moderate hypothermia (32–34 °C) was associated with a decreased number of apoptotic hippocampal neurons [54]. These experimental elements were confirmed by other studies, which made it possible to specify the involved pathophysiological mechanisms. Yu et al. showed that moderate hypothermia (32–34 °C) was associated with regulation of the expression of the GluR1 and GluR2 subunits of α-amino-3-hydroxy-5-methyl-4-isoxazolepropionic acid (AMPA) receptors to glutamate, again reducing the volume of necrotic hippocampal neurons and apoptosis [55]. More recently, Phillips et al. showed a decrease in intracellular calcium entry via the activity of N-methyl-d-aspartate (NMDA) receptors in rats receiving hypothermia [56]. Finally, Wang et al. also made an important contribution to our understanding of the neurotoxicity associated with SE by demonstrating a decrease in brain edema induced by seizure activity and improved cognitive abilities in rats treated by hypothermia immediately after an episode of SE induced by kainic acid [54]. 

#### 3.1.5. Natremia/Osmolality

The regulation of changes in osmolarity is mediated by osmoreceptors localized in the hypothalamus and involves the supraoptic and paraventricular nuclei of the hypothalamus neurons, which synthesize the antidiuretic hormone that acts at the posterior lobe of the pituitary gland. Changes in osmolarity can be determined by direct measurement of plasma osmolality or calculated according to the following formula: osmolarity = (2 × natremia (mmol/L)) + glycemia (mmol/L) + uremia (mmol/L). Thus, any sudden changes in osmolarity related to the variation of one of its determinants (most often natremia) can affect intracellular hydration and, consequently, neuronal intracellular tonicity. The blood brain barrier (BBB) is permeable to water and small lipophilic molecules, but impervious to electrolytes and plasma proteins. Small changes in plasma osmolarity, therefore, cause significant movement of water on both sides of this barrier. Natremia is one of the main determinants of plasma osmolarity, modulating the movement of water between plasma and the cerebral parenchyma. Thus, hypo-osmolar hyponatremia leads to plasma hypo-osmolarity and the transfer of water from the plasma to brain cells, leading to an increase in intracranial pressure, the appearance of cerebral edema, and intracranial hypertension. Conversely, hypernatremia leads to plasma hyperosmolarity and the movement of water towards the plasma sector, with a decrease in cerebral edema and ICP. However, brain cells have a mechanism to regulate osmolarity, which makes it possible to limit the variations in their volume in either situation.

We found no study evaluating the direct association of natremia and seizures or SE in animals. However, several studies performed on hippocampal tissue slices tested the effects of osmolality changes on neuronal activity; they demonstrated the enhancement of epileptiform discharges with decreasing osmolality. Conversely, any increase in osmolality resulted in less neuronal epileptiform activity [57,58,59,60]. These in vitro findings were confirmed in animal studies. Indeed, adult rats receiving hypo-osmolar treatment by intraperitoneal administration of distilled water demonstrated high BBB permeability [61]. Similar alterations in BBB integrity were observed after epileptic seizures [62]. 

#### 3.1.6. Glycemia

Moderate hypoglycemia was demonstrated to be associated with BBB alterations in an experimental study on Wistar rats subjected to electroconvulsive seizures [63]. The severity of BBB breakdown was linked to the number of electroconvulsive seizures. Such alterations occurred mostly in the thalamus, hypothalamus, amygdale nuclei, and frontoparietal and occipital cortices. Conversely, rats exposed to hyperglycemia and seizures exhibited no significant BBB dysfunction relative to normoglycemic rats [63,64]. Finally, further experimental findings on mice demonstrated a relationship between dysglycemia and seizure-induced cell death. In these animals, glycemic control might participate in diminishing neuronal injury [65]. 

Hypoglycemia has also been demonstrated to be responsible for clinical seizure activity. The involved pathophysiological mechanisms are related to aspartate release to the extracellular space of the brain, increasing the levels of excitatory amino acids responsible for seizure activity [66]. Paradoxically, in vitro glucose depletion contributes to the arrest of epileptiform activity [67]. The reason for this discrepancy between clinical and in vitro findings has yet to be elucidated.

Hyperglycemia also has proconvulsant properties. Higher glucose levels triggered epileptiform activity in an experimental study. Indeed, in vitro epileptiform activity correlated with glucose levels, and the lower epileptic threshold was associated with a higher glucose concentration, whereas a lower a glucose concentration was suppressive [64]. 

#### 3.1.7. Hemoglobinemia

Arterial oxygen transport depends on oxygen (O_2_) and hemoglobinemia. Thus, hemoglobin plays an important role in increasing the cerebral tissue pressure in oxygen (PtiO_2_), which is the true marker of cerebral oxygenation. However, anemia decreases blood viscosity and is associated with an increase in CBF. Its role as a factor in secondary brain insults is therefore a subject of debate in traumatic brain injury. There has been no reported association of anemia and seizures or SE in animals.

### 3.2. Clinical Applications of Secondary Brain Insults in the Management of Status Epilepticus

Physiologic changes linked to ictal activity were investigated in few clinical studies. In patients with subarachnoid hemorrhage, nonconvulsive seizures were independently associated systemic inflammatory response syndrome characterized by higher tumor necrosis factor receptor and high-sensitivity C-reactive protein serum levels [68]. Moreover, SE is associated with systemic complications that may lead to multiple organ failure and death. Most of them can directly or indirectly be responsible for the secondary brain insults cited above. For instance, cardiac complications, which can be encountered in two-thirds of patients with SE, may be associated by arterial blood pressure hypotension. Similarly, respiratory failure, occurring in about one-third of cases during SE, may induce hypocapnia, hypercapnia, and hypoxemia [69]. 

However, apart from one randomized controlled trial that assessed the effect of targeted temperature management, no strategy specifically targeted to the management of secondary brain insults in SE has been investigated to date. In this trial, a group of patients with convulsive SE, requiring mechanical ventilation for refractory convulsive SE or any other reason, receiving therapeutic hypothermia treatment (between 32 and 34 °C) were compared to a similar group of patients receiving normothermic treatment. The primary endpoint was neuroprotection, assessed by a 90 day Glasgow Outcome Scale score of 5, corresponding to a good recovery, in other words, full recovery or minor symptoms that do not affect daily life. The secondary endpoints were concerned with evaluation of the antiepileptic properties of therapeutic hypothermia, namely total seizure duration and progression to non-convulsive SE, refractory SE, or super refractory SE [70]. The results were negative for the primary endpoint, as 49% of patients in the hypothermia group demonstrated a 90 day GOS of 5 versus 43% in the normothermia group. The results for the secondary endpoints showed a favorable trend of therapeutic hypothermia for an anticonvulsant effect. Progression to non-convulsive SE was the only variable that significantly decreased in the hypothermia group relative to the normothermia group [70]. Finally, the results of this randomized controlled trial did not support the use of therapeutic hypothermia as a neuroprotective strategy after convulsive status epilepticus but provided encouraging data regarding its anticonvulsant activity.

## 4. Perspectives

SE is associated with various physiologic changes and complications that can lead to secondary brain insults, as consequences and/or sequelae of ongoing seizure activity, but also as consequences and/or sequalae of the treatments of refractory or super refractory SE, as well as the underlying critical illness which further exacerbates and contributes to secondary brain insults. Further studies are required to evaluate the association of secondary brain insults and cause of death in status epilepticus, and to assess the neuroprotective benefit of controlling secondary brain insult after SE [71].

## 5. Conclusions

There is little experimental evidence for the control of secondary brain insult after SE. In humans, only target temperature management in hypothermia (32–34 °C) has been explored. Although a neuroprotective effect of hypothermia failed to be demonstrated, target temperature management between 32 and 34 °C demonstrated promising anticonvulsant effects in patients with convulsive SE.

## References

[1] Holleville M., Jacq G., Perier F., Fontaine C., Legriel S. (2020). Epileptic Seizures in Critically Ill Patients: Diagnosis, Management, and Outcomes. J. Clin. Med..

[2] Legriel S., Mourvillier B., Bele N., Amaro J., Fouet P., Manet P., Hilpert F. (2008). Outcomes in 140 critically ill patients with status epilepticus. Intensive Care Med..

[3] Legriel S., Azoulay E., Resche-Rigon M., Lemiale V., Mourvillier B., Kouatchet A., Troche G., Wolf M., Galliot R., Dessertaine G. (2010). Functional outcome after convulsive status epilepticus. Crit. Care Med..

[4] Werner C., Engelhard K. (2007). Pathophysiology of traumatic brain injury. Br. J. Anaesth..

[5] Siesjo B.K., Siesjo P. (1996). Mechanisms of secondary brain injury. Eur. J. Anaesthesiol..

[6] Vaughan C.J., Delanty N. (2000). Hypertensive emergencies. Lancet.

[7] Geeraerts T., Velly L., Abdennour L., Asehnoune K., Audibert G., Bouzat P., Bruder N., Carrillon R., Cottenceau V., Cotton F. (2018). Management of severe traumatic brain injury (first 24 hours). Anaesth. Crit. Care Pain Med..

[8] Chesnut R.M., Marshall L.F., Klauber M.R., Blunt B.A., Baldwin N., Eisenberg H.M., Jane J.A., Marmarou A., Foulkes M.A. (1993). The role of secondary brain injury in determining outcome from severe head injury. J. Trauma.

[9] Carney N., Totten A.M., O’Reilly C., Ullman J.S., Hawryluk G.W., Bell M.J., Bratton S.L., Chesnut R., Harris O.A., Kissoon N. (2017). Guidelines for the Management of Severe Traumatic Brain Injury, Fourth Edition. Neurosurgery.

[10] Cariou A., Payen J.F., Asehnoune K., Audibert G., Botte A., Brissaud O., Debaty G., Deltour S., Deye N., Engrand N. (2017). Targeted temperature management in the ICU: Guidelines from a French expert panel. Ann. Intensive Care.

[11] Ntaios G., Dziedzic T., Michel P., Papavasileiou V., Petersson J., Staykov D., Thomas B., Steiner T., European Stroke O. (2015). European Stroke Organisation (ESO) guidelines for the management of temperature in patients with acute ischemic stroke. Int. J. Stroke.

[12] Bederson J.B., Connolly E.S., Batjer H.H., Dacey R.G., Dion J.E., Diringer M.N., Duldner J.E., Harbaugh R.E., Patel A.B., Rosenwasser R.H. (2009). Guidelines for the management of aneurysmal subarachnoid hemorrhage: A statement for healthcare professionals from a special writing group of the Stroke Council, American Heart Association. Stroke.

[13] Meldrum B.S., Horton R.W. (1973). Physiology of status epilepticus in primates. Arch. Neurol..

[14] Walton N.Y. (1993). Systemic effects of generalized convulsive status epilepticus. Epilepsia.

[15] Wasterlain C.G., Fujikawa D.G., Penix L., Sankar R. (1993). Pathophysiological mechanisms of brain damage from status epilepticus. Epilepsia.

[16] Trinka E., Cock H., Hesdorffer D., Rossetti A.O., Scheffer I.E., Shinnar S., Shorvon S., Lowenstein D.H. (2015). A definition and classification of status epilepticus--Report of the ILAE Task Force on Classification of Status Epilepticus. Epilepsia.

[17] Aminoff M.J., Simon R.P. (1980). Status epilepticus. Causes, clinical features and consequences in 98 patients. Am. J. Med..

[18] Simon R.P. (1985). Physiologic consequences of status epilepticus. Epilepsia.

[19] Posner J.B., Plum F. (1968). Cerebral metabolism during electrically-induced seizures in man. Trans. Am. Neurol. Assoc..

[20] Meldrum B.S., Nilsson B. (1976). Cerebral blood flow and metabolic rate early and late in prolonged epileptic seizures induced in rats by bicuculline. Brain.

[21] Ingvar M., Siesjo B.K. (1983). Local blood flow and glucose consumption in the rat brain during sustained bicuculline-induced seizures. Acta Neurol. Scand..

[22] Blennow G., Brierley J.B., Meldrum B.S., Siesjo B.K. (1978). Epileptic brain damage: The role of systemic factors that modify cerebral energy metabolism. Brain.

[23] Laffey J.G., Kavanagh B.P. (2002). Hypocapnia. N. Engl. J. Med..

[24] Holmes M.D., Dewaraja A.S., Vanhatalo S. (2004). Does hyperventilation elicit epileptic seizures?. Epilepsia.

[25] Bergsholm P., Gran L., Bleie H. (1984). Seizure duration in unilateral electroconvulsive therapy. The effect of hypocapnia induced by hyperventilation and the effect of ventilation with oxygen. Acta Psychiatr. Scand..

[26] Marrosu F., Puligheddu M., Giagheddu M., Cossu G., Piga M. (2000). Correlation between cerebral perfusion and hyperventilation enhanced focal spiking activity. Epilepsy Res..

[27] Sawayama E., Takahashi M., Inoue A., Nakajima K., Kano A., Sawayama T., Okutomi T., Miyaoka H. (2008). Moderate hyperventilation prolongs electroencephalogram seizure duration of the first electroconvulsive therapy. J. ECT.

[28] Jonas J., Vignal J.P., Baumann C., Anxionnat J.F., Muresan M., Vespignani H., Maillard L. (2011). Effect of hyperventilation on seizure activation: Potentiation by antiepileptic drug tapering. J. Neurol. Neurosurg. Psychiatry.

[29] Balestrino M., Somjen G.G. (1988). Concentration of carbon dioxide, interstitial pH and synaptic transmission in hippocampal formation of the rat. J. Physiol..

[30] Crawford C.D., Butler P., Froese A. (1987). Arterial PaO2 and PaCO2 influence seizure duration in dogs receiving electroconvulsive therapy. Can. J. Anaesth..

[31] Dulla C.G., Dobelis P., Pearson T., Frenguelli B.G., Staley K.J., Masino S.A. (2005). Adenosine and ATP link PCO2 to cortical excitability via pH. Neuron.

[32] Ziemann A.E., Schnizler M.K., Albert G.W., Severson M.A., Howard Iii M.A., Welsh M.J., Wemmie J.A. (2008). Seizure termination by acidosis depends on ASIC1a. Nat. Neurosci..

[33] Legriel S., Mentec H. (2005). Status epilepticus during acute hypercapnia: A case report. Intensive Care Med..

[34] Brodie D.A., Woodbury D.M. (1958). Acid-base changes in brain and blood of rats exposed to high concentrations of carbon dioxide. Am. J. Physiol..

[35] Withrow C.D., Nord N.M., Turner L.M., Woodbury D.M. (1967). Carbon dioxide seizures in immature rats. Proc. Soc. Exp. Biol. Med..

[36] Katsura K., Kristian T., Smith M.L., Siesjo B.K. (1994). Acidosis induced by hypercapnia exaggerates ischemic brain damage. J. Cereb. Blood Flow Metab..

[37] Mathern G.W., Price G., Rosales C., Pretorius J.K., Lozada A., Mendoza D. (1998). Anoxia during kainate status epilepticus shortens behavioral convulsions but generates hippocampal neuron loss and supragranular mossy fiber sprouting. Epilepsy Res..

[38] Aksay S.S., Bumb J.M., Janke C., Hoyer C., Kranaster L., Sartorius A. (2014). New evidence for seizure quality improvement by hyperoxia and mild hypocapnia. J. ECT.

[39] Wasterlain C.G. (1974). Mortality and morbidity from serial seizures. An experimental study. Epilepsia.

[40] Nevander G., Ingvar M., Auer R., Siesjo B.K. (1985). Status epilepticus in well-oxygenated rats causes neuronal necrosis. Ann. Neurol..

[41] Amano S., Obata T., Hazama F., Kashiro N., Shimada M. (1990). Hypoxia prevents seizures and neuronal damages of the hippocampus induced by kainic acid in rats. Brain Res..

[42] Emerson M.R., Nelson S.R., Samson F.E., Pazdernik T.L. (1999). Hypoxia preconditioning attenuates brain edema associated with kainic acid-induced status epilepticus in rats. Brain Res..

[43] Elayan I.M., Axley M.J., Prasad P.V., Ahlers S.T., Auker C.R. (2000). Effect of hyperbaric oxygen treatment on nitric oxide and oxygen free radicals in rat brain. J. Neurophysiol..

[44] Lambertsen C., Clark J., Gelfand R., Pisarello J., Cobbs W. Definition of tolerance to continuous hyperoxia in man-An abstract report of Predictive Studies V. Proceedings of the 9th International Symposium on Underwater and Hyperbaric Physiology.

[45] Soderfeldt B., Blennow G., Kalimo H., Olsson Y., Siesjo B.K. (1983). Influence of systemic factors on experimental epileptic brain injury. Structural changes accompanying bicuculline-induced seizures in rats following manipulations of tissue oxygenation or alpha-tocopherol levels. Acta Neuropathol..

[46] Pomper J.K., Hoffmann U., Kovacs R., Gabriel S., Heinemann U. (2004). Hyperoxia is not an essential condition for status epilepticus induced cell death in organotypic hippocampal slice cultures. Epilepsy Res..

[47] Cungi P.J., Holleville M., Fontaine C., Jacq G., Legriel S. (2020). Second-line anticonvulsant for convulsive status epilepticus: The dosage matters!. Anaesth. Crit. Care Pain Med..

[48] Jiang W., Duong T.M., de Lanerolle N.C. (1999). The neuropathology of hyperthermic seizures in the rat. Epilepsia.

[49] Liu Z., Gatt A., Mikati M., Holmes G.L. (1993). Effect of temperature on kainic acid-induced seizures. Brain Res..

[50] Lundgren J., Smith M.L., Blennow G., Siesjo B.K. (1994). Hyperthermia aggravates and hypothermia ameliorates epileptic brain damage. Exp. Brain Res..

[51] Maeda T., Hashizume K., Tanaka T. (1999). Effect of hypothermia on kainic acid-induced limbic seizures: An electroencephalographic and 14C-deoxyglucose autoradiographic study. Brain Res..

[52] Schmitt F.C., Buchheim K., Meierkord H., Holtkamp M. (2006). Anticonvulsant properties of hypothermia in experimental status epilepticus. Neurobiol. Dis..

[53] Kowski A.B., Kanaan H., Schmitt F.C., Holtkamp M. (2012). Deep hypothermia terminates status epilepticus--an experimental study. Brain Res..

[54] Wang Y., Liu P.P., Li L.Y., Zhang H.M., Li T. (2011). Hypothermia reduces brain edema, spontaneous recurrent seizure attack, and learning memory deficits in the kainic acid treated rats. CNS Neurosci. Ther..

[55] Yu L., Zhou Y., Wang Y. (2012). Effect of mild hypothermia on glutamate receptor expression after status epilepticus. Epilepsy Res..

[56] Phillips K.F., Deshpande L.S., DeLorenzo R.J. (2013). Hypothermia reduces calcium entry via the N-methyl-D-aspartate and ryanodine receptors in cultured hippocampal neurons. Eur. J. Pharmacol..

[57] Dudek F.E., Obenaus A., Tasker J.G. (1990). Osmolality-induced changes in extracellular volume alter epileptiform bursts independent of chemical synapses in the rat: Importance of non-synaptic mechanisms in hippocampal epileptogenesis. Neurosci. Letters.

[58] Huang R., Somjen G.G. (1997). Effects of hypertonia on voltage-gated ion currents in freshly isolated hippocampal neurons, and on synaptic currents in neurons in hippocampal slices. Brain Res..

[59] Andrew R.D., Fagan M., Ballyk B.A., Rosen A.S. (1989). Seizure susceptibility and the osmotic state. Brain Res..

[60] Roper S.N., Obenaus A., Dudek F.E. (1992). Osmolality and nonsynaptic epileptiform bursts in rat CA1 and dentate gyrus. Ann. Neurol..

[61] Oztas B., Kaya M., Kucuk M., Tugran N. (2003). Influence of hypoosmolality on the blood-brain barrier permeability during epileptic seizures. Prog. Neuro. Psychopharmacol. Biol. Psychiatry.

[62] Janigro D. (1999). Blood-brain barrier, ion homeostatis and epilepsy: Possible implications towards the understanding of ketogenic diet mechanisms. Epilepsy Res..

[63] Oztas B., Camurcu S. (1989). Blood-brain barrier permeability after electrically induced seizure in normoglycemic, hypoglycemic, and hyperglycemic rats. Psychiatry Res..

[64] Schwechter E.M., Veliskova J., Velisek L. (2003). Correlation between extracellular glucose and seizure susceptibility in adult rats. Ann. Neurol..

[65] Schauwecker P.E. (2012). The effects of glycemic control on seizures and seizure-induced excitotoxic cell death. BMC Neurosci..

[66] Auer R.N. (2004). Hypoglycemic brain damage. Forensic Sci. Int..

[67] Kirchner A., Veliskova J., Velisek L. (2006). Differential effects of low glucose concentrations on seizures and epileptiform activity in vivo and in vitro. Eur. J. Neurosci..

[68] Claassen J., Albers D., Schmidt J.M., De Marchis G.M., Pugin D., Falo C.M., Mayer S.A., Cremers S., Agarwal S., Elkind M.S. (2014). Nonconvulsive seizures in subarachnoid hemorrhage link inflammation and outcome. Ann. Neurol..

[69] Hawkes M.A., Hocker S.E. (2018). Systemic Complications Following Status Epilepticus. Curr. Neurol. Neurosci. Rep..

[70] Legriel S., Lemiale V., Schenck M., Chelly J., Laurent V., Daviaud F., Srairi M., Hamdi A., Geri G., Rossignol T. (2016). Hypothermia for Neuroprotection in Convulsive Status Epilepticus. N. Engl. J. Med..

[71] Hawkes M.A., English S.W., Mandrekar J.N., Rabinstein A.A., Hocker S. (2019). Causes of Death in Status Epilepticus. Crit. Care Med..

